# The Role of Biologically Active Ingredients from Natural Drug Treatments for Arrhythmias in Different Mechanisms

**DOI:** 10.1155/2017/4615727

**Published:** 2017-04-11

**Authors:** Jie Li, Dan Hu, Xiaoli Song, Tao Han, Yonghong Gao, Yanwei Xing

**Affiliations:** ^1^Guang'anmen Hospital, Chinese Academy of Chinese Medical Sciences, Beijing 100053, China; ^2^Masonic Medical Research Laboratory, Utica, NY, USA; ^3^Shandong University of Traditional Chinese Medicine, Jinan 250355, China; ^4^Key Laboratory of Chinese Internal Medicine of the Ministry of Education, Dongzhimen Hospital Affiliated to Beijing University of Chinese Medicine, Beijing 100700, China

## Abstract

Arrhythmia is a disease that is caused by abnormal electrical activity in the heart rate or rhythm. It is the major cause of cardiovascular morbidity and mortality. Although several antiarrhythmic drugs have been used in clinic for decades, their application is often limited by their adverse effects. As a result, natural drugs, which have fewer side effects, are now being used to treat arrhythmias. We searched for all articles on the role of biologically active ingredients from natural drug treatments for arrhythmias in different mechanisms in PubMed. This study reviews 19 natural drug therapies, with 18 active ingredient therapies, such as alkaloids, flavonoids, saponins, quinones, and terpenes, and two kinds of traditional Chinese medicine compound (Wenxin-Keli and Shensongyangxin), all of which have been studied and reported as having antiarrhythmic effects. The primary focus is the proposed antiarrhythmic mechanism of each natural drug agent.* Conclusion*. We stress persistent vigilance on the part of the provider in discussing the use of natural drug agents to provide a solid theoretical foundation for further research on antiarrhythmia drugs.

## 1. Introduction

Cardiac arrhythmia is a disease that is caused by abnormal electrical activity in the heart rate or rhythm. It has been shown that 88% of sudden cardiac deaths are caused by cardiac arrhythmia, which results in the development of serious complications in the heart and other organ diseases [1]. Western medicine plays an important role in therapeutic approaches to arrhythmia. To inhibit irregular electrical activities, antiarrhythmic drugs act by targeting the cardiomyocyte membrane ion channel, altering the conduction velocity, and repressing trigger activity. The conventional treatments for acute arrhythmia are not only single action treatments, but also limited and prone to side effects. But beyond that, they have potential proarrhythmic effects [2]. Recently, some studies found that the therapeutic effects of Western medicines are far from satisfactory. There is a medicine called amiodarone, which is the most widely used drug for cardiac arrhythmia currently. Amiodarone reportedly has serious side effects, such as thyrotoxicity, pulmonary fibrosis, and liver damage [3].

Previous clinical and experimental investigations have indicated that natural drugs can inhibit the occurrence of arrhythmia to some extent [4]. These drugs can not only block cardiac ion channels and regulate the cardiac autonomic nerve, quickening the treatment of various cardiac arrhythmias, but can also enhance the cardiac pacemaker current, improving heart function. Natural drugs block cardiac arrhythmias through multiple pathways, target points, and elements. Natural drugs as well as their purified forms have efficient antiarrhythmic actions; furthermore, their effects are permanent and steady, and their toxicity and side effects are low [5, 6]. We then summarized the recent advances on the pharmacological effects of natural antiarrhythmic drugs in treating cardiac arrhythmia, exploring their potential mechanisms, and looking for novel targets.

**Figure 1:**
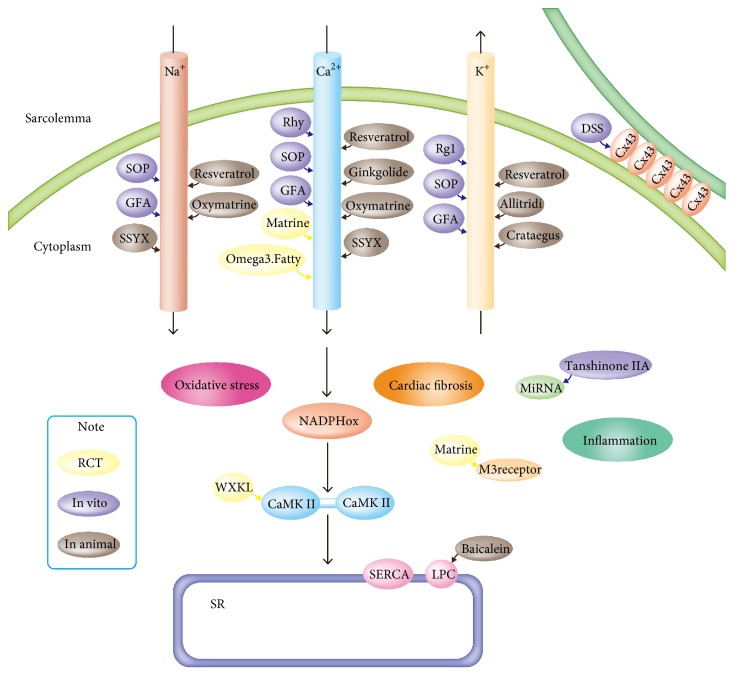
Schematic overview of the proposed antiarrhythmic mechanism of each natural drug agent. SR = sarcoplasmic reticulum. SERCA = SR Ca^2+^ATPase.

*Natural Drug Agents with Reported Antiarrhythmic Properties*. A list of these natural drugs, their proposed mechanisms, and known natural drug interactions is presented in Table 1 and Figure 1.

## 2. Alkaloids

### 2.1. Diterpenoid Alkaloids

Aconitine was found in Radix Aconiti (Kusnezoff Monkshood) roots, and the aconitine in the lateral roots is the primary toxic ingredient in these plants. Pharmacological studies showed that aconitine had antiarrhythmic and analgesic effects, as well as anti-inflammatory activities. This herb can be used for the isolation of several diterpenoid alkaloids (DAs). Guan-fu base A (GFA, 16), one of the dominant DAs in this herb, has been developed into a new antiarrhythmia drug, and it has also been used in clinical medicine [7]. There is a large body of research showing that Guan-fu base A (GFA) blocks the fast Na+ channel (INa), slowly activated delayed rectifier potassium current (Iks), and the L-type calcium channel (I_Ca-L_) [8]. Recently, more and more studies have demonstrated that Guan-fu bases (GFS, 3) can inhibit the sodium channel current. This finding suggests that GFS (3) is a promising antiarrhythmia agent [9].

### 2.2. Isoquinoline

#### 2.2.1. Berberine

Berberine is an important isoquinoline alkaloid, and it can be extracted from* Coptis chinensis* Franch [10]. It has very extensive pharmacological activities, such as antibacterial, antipyretic, antipruritic, and antiarrhythmic activities. Its antiarrhythmic effect is one of its most remarkable activities [11]. Previous pharmacologic studies on berberine showed that it possesses potent vasodilatory and antiarrhythmic activity. It may prolong the action potential duration (APD) of antiarrhythmic activity through the dose-dependent inhibition of I_to_. This type of effect is similar to the antiarrhythmic effects of disopyramide and quinidine, and its activity has been shown in animal models and human atrial cells in vitro. The berberine of the I_to_ blockade is different from the disopyramide and quinidine because it is not accompanied by an inward sodium current inhibition [12]. Another research has shown that it may lead to the prolongation of the QT interval and increase the risk of ventricular arrhythmias [13]. Furthermore, we used voltage clamp operations to observe that berberine had significant inhibitory effects that delayed the potassium current, and not the sodium current, which is the extension of the myocardial cells and one of the important mechanisms underlying antiarrhythmia.

#### 2.2.2. Liensinine

Liensinine is a type of isoquinoline alkaloid. It is present in the Nymphaeaceae germs of lotus seed plants (called lotus nuts), and the lotus nut is a common Chinese herbal medicine. Liensinine has documented beneficial pharmacological effects, such as antioxidant, antiarrhythmic, and antihypertension effects, and it causes the relaxation of vascular smooth muscles [14]. Recently, various studies have indicated that liensinine could block the L-type calcium channel current and prolong the action potential duration. The human ether-a-go-go-related gene (hERG) has become an important target of antiarrhythmia therapy. Research shows that liensinine can inhibit the hERG tail current in a dose-dependent manner. Both liensinine and neferine were potential hERG channel blockers. Liensinine can resist the ventricular arrhythmias [15].

### 2.3. Indole Alkaloids

Rhynchophylline (Rhy) is a major tetracyclic oxindole alkaloid that is isolated from* Uncaria* species. Rhynchophylline was traditionally used to treat headaches, vertigo, and epilepsy [16]. In recent years, Rhy has been shown to possess multiple pharmacological activities, such as antiarrhythmic, antihypertensive, neuroprotective, and anti-inflammatory effects [17]. Recent studies indicate that isorhynchophylline could significantly decrease the action potential duration, and it could inhibit calcium currents in isolated guinea pig and rat cardiomyocytes in a dose-dependent manner [18].

### 2.4. Quinolizidine Alkaloids

#### 2.4.1. Matrine

Matrine is an alkaloid that is extracted from the* Sophora* (dry bean plant) root. It is extracted by using organic solvents such as ethanol. Recent experimental studies have identified it as the bioactive component that contributes to a variety of pharmacological effects, such as hepatitis B and C, cancer, and cardiac disease activities [19]. In clinics, matrine is currently used to treat cardiac arrhythmias, especially premature ventricular beats. Based on previous research, investigators found that matrine possessed antiarrhythmic effects in experimental arrhythmic models that were induced by coronary artery ligation and electric stimulation in rats and rabbits. Matrine could inhibit K^+^ channels (I_KM3_) and prolong APD. More importantly, some studies have found that it prolonged repolarization and increased the effective refractory period (ERP) of the myocardium [20]. The present study was designed to stimulate L-type calcium channels (I_Ca-L_) to reduce Ca^2+^ overload [21].

#### 2.4.2. Oxymatrine

Oxymatrine is separated from* Sophora flavescens* or* Sophora subprostrata*, and it is one of the primary quinolizidine alkaloids [22]. Researchers have reported that it has a wide scope of cardiovascular pharmacological effects, including antiarrhythmia, antihypertension, and antiventricular remodeling as well as antimyocardial fibrosis [23]. Electrophysiological studies indicated that oxymatrine could inhibit sodium and calcium currents in isolated rat cardiomyocytes in a concentration-dependent manner. Furthermore, oxymatrine significantly delayed the initial time and shortened the duration time of rat arrhythmias induced by coronary artery ligation [24].

#### 2.4.3. Sophocarpine

Sophocarpine (SOP) is a dehydrogenation derivative of matrine. It is extracted from a traditional Chinese medicine* (Sophora flavescens)* that has been used for centuries [25]. Pharmacology experiments have verified that it has anti-inflammation, antivirus, antitumor, immune adjustment, and cardiovascular disease prevention functions [26]. Above all, it has been shown to work effectively against heart diseases [27], particularly in the treatment of viral myocarditis and ventricular arrhythmias. Researchers suggest that SOP can inhibit the Na^+^ current (I_Na_), L-type calcium current (I_CaL_), and potassium current (I_Kr_) to reverse isoprenaline-induced arrhythmia. In addition, other studies have also shown that it could inhibit the human ether-a-go-go-related gene (hERG) potassium channel, prolong myocardial APD, and improve tachyarrhythmia in a concentration-dependent manner [28]. Another research has demonstrated that SOP was similar to tetrodotoxin (TTX), and it inhibits the I_Na_,  I_NCX_, and diastolic Ca^2+^ concentration, in addition to contractility in rabbit ventricular myocytes. It may become a new therapeutic mechanism for SOP against arrhythmia and the myocyte damage associated with intracellular Ca^2+^ overload [29].

## 3. Glycosides

### 3.1. Ginsenoside

Ginsenoside is a type of sterol compound, or triterpenoid saponin, which is primarily present in* Panax* medicinal materials. Ginsenoside is known as the active ingredient in ginseng, making it the target of many studies. Ginsenoside Rg1 (Rg1) is one of the most active ingredients in* Panax ginseng*. It has been used frequently in relation to cardiovascular diseases; for instance, Rg1 can reduce the ventricular remodeling induced by myocardial infarction [30] and the left ventricular hypertrophy induced by abdominal aorta coarctation in rats [31]. Electrophysiological experiments showed that ginsenoside Rg1 could prolong ventricular refractoriness and repolarization, and it could increase the ventricular fibrillation threshold. The cardiac electrophysiological effects of Rg1 were also said to be similar to those of amiodarone [32]. Other reports suggested that ginsenoside Rg1 increased the outward hERG current, and it provided potential protection against myocardial infarction [33].

### 3.2. Phenylpropanoid Glycosides

Motherwort* (L. cardiaca)* has a long history of use in both European and Asian traditional medicine. It has been used in traditional medicine for nervous and functional cardiac disorders since the 15th century [34]. It is now described in pharmacopoeias for producing sedative, hypotensive, and cardiotonic pharmacological effects. Phenylpropanoid glycosides have been discovered in* Leonurus glaucescens* [35]. The pharmacological activities of these compounds have shown that they can decrease the frequency of the isolated rat heart, prolong the PQ intervals, lengthen the duration of PQ, QT intervals, and diminish the coronary outflow [36]. In addition, voltage clamp measurements were performed to show that phenylpropanoid glycosides significantly inhibited the L-type calcium current, reduced the repolarizing current IKr, and prolonged the AP duration [37].

## 4. Flavonoids

### 4.1. Baicalein

Baicalein is extracted from* Scutellaria baicalensis* Georgi. It has a variety of biological activities; that is, it has antithrombotic, antiviral, anticancer, and anti-inflammatory activities. Previous studies indicated that lysophosphatidylcholine (Pal-LPC) can clearly change the potassium channel current activities, which affect the occurrence of arrhythmia [38]. Recent studies show that, after being treated with baicalein, the contractile function of the isolated heart was significantly preserved for 6 h after LPS administration [39].

### 4.2. Resveratrol

Resveratrol is a polyphenol compound that is primarily derived from grapes (red wine), giant knotweed, peanuts, mulberries, and other plants. Resveratrol is considered as a more effective antioxidant and is more bioactive [40]. Other studies have revealed that resveratrol could shorten the action potential duration through I_Ca_ inhibition and the selective enhancement of I_Ks_ without having an effect on I_Kr_ [41]. Electrophysiological experiments have attributed the inhibition of L-type Ca^2+^ channels by resveratrol to the inhibition of protein tyrosine kinase in rat cardiomyocytes [42]. However, researchers have shown that resveratrol could inhibit inward sodium and calcium currents and increase the cardiac refractory period [43]. Similarly, other researchers have shown that resveratrol inhibits oxidative stress-induced arrhythmogenic activity in rabbit ventricular myocytes by inhibiting late Na^+^ current and L-type Ca^2+^ current.

## 5. Terpenes

The medicinal use of* Ginkgo biloba* can be traced back almost 5,000 years in Chinese herbal medicine [44]. Ginkgolide is extracted from the leaves of* Ginkgo biloba*, and it has been used therapeutically for dementia and Alzheimer's disease, and for peripheral vascular diseases such as arterial occlusive disease [45]. The pharmacological mechanisms have already shown that ginkgolide could inhibit the L-type Ca^2+^ current (I_Ca_) and the hyperpolarization-activated inward current (I_f_) [46]. Recent clinical and experimental work has shown that ginkgolide shortens the APD (action potential duration) and inhibits the L-type calcium currents in isolated guinea pig ventricular myocytes. These results indicate that ginkgolide (GLD) can prevent ischemic arrhythmias and have a potential antiarrhythmic effect [47].

## 6. Quinones

Tanshinone was extracted from the traditional Chinese medicine danshen (*Salvia miltiorrhiza,* Labiatae,* Salvia miltiorrhiza* Bunge) root. This compound causes the same bacteriostasis as fat-soluble phenanthrene quinone compounds. It can be divided into Tanshinone* I*, Tanshinone* II*A, and so forth. Tanshinone* II*A is an active component from* Salvia miltiorrhiza* that is used to suppress ischemic arrhythmias [48]. According to investigators, Tanshinone* II*A restored the diminished I_K1_ current density and K_ir2.1_ protein after MI in rat ventricular myocytes [49]. These results indicate that Tanshinone* II*A potently and specifically enhances I_Ks_ by affecting the channel kinetics, and its effect is independent of protein kinase A (PKA) activation, protein kinase G (PKG) activation, and channel nitrosylation. These studies also show that Tanshinone* II*A can reduce overexpressed miR-1 by regulating SRF. It can also significantly improve the myocardial tissue content (IMA and H-FABP) [50]. Therefore, miR-1 could be a potential therapeutic target for the prevention of ischemic arrhythmias.

## 7. Others

### 7.1. *Crataegus*

*Crataegus* is extracted from the berries and flowers of the common hawthorn plant. Its use as a cardiovascular agent in European medicine dates to the 1st century Greek herbalist Dioscorides and the Swiss physician Paracelsus (1493 to 1541). Studies on receiver biases suggest that this compound can be used to treat angina, arrhythmia, hypertension, and congestive heart failure [51]. Much of the research on this drug over the last two decades has shown that it can inhibit the inward potassium channels I_Ks_ and I_Kr_. It can also prolong the action potential [52].* Crataegus* extract increases the action potential duration. This effect is roughly identical to that of class 3 antiarrhythmic agents, and it provides the basis for the antiarrhythmic effects described for hawthorn extract [53]. Even more importantly, the selective nature of the* Crataegus* effects differentiates it from class 3 agents in that it is not accompanied by additional *β*- or calcium channel-blocking properties [54].

### 7.2. Danshensu

Danshensu (DSS) is an active water-soluble component from* Salvia miltiorrhiza* (Labiatae) plants (*Salvia miltiorrhiza*). The gap junction protein (Cx43) is one of the most basic proteins between a myocardial cell passage and gap junctions. It plays an important role in myocardial ischemia, reperfusion injury, and the progress of intercalated disc refactoring [55]. Recent reports indicate that DSS could reverse downregulated Cx43 protein levels, and it shows potent antioxidative activities and provides cellular protection [56]. DSS can effectively inhibit I/R arrhythmias in hypertrophy-*induced* rats through l-thy, preventing hypertrophy progression in rats.

### 7.3. Omega-3 Fatty Acids

Omega-3 fatty acids are polyunsaturated fatty acids that are found in deep-sea fishes and certain plants. Since the 1970s, scientists have discovered that people living in Greenland rarely experience cardiovascular disease, and investigators gradually began to research omega-3 fatty acids in depth [57]. Scientists from all over the world have addressed the omega-3 fatty acids in the study, and more than 15000 studies show that omega-3 fatty acids have anti-inflammatory actions and help in resisting thrombosis, reducing blood fat, and maintaining healthy blood vessels and the resting heart rate if correct [58]. Many clinical investigations have demonstrated that it has significant antiarrhythmic properties. Numerous additional studies have supported the benefit of fish oil intake for reducing serious ventricular arrhythmias [59]. Most experimental studies indicate that omega-3 fatty acids can prevent or attenuate *β* agonist-induced arrhythmias in vitro, possibly supporting a *β* blockade-like effect [60]. In addition, omega-3 fatty acids have been shown to prevent fatal ventricular arrhythmias, and they can prevent calcium overload during stress by inhibiting voltage-gated sodium channels and maintaining L-type calcium channels [61].

### 7.4. Allitridi

*Allium sativum* L. (Da-Suan in Mandarin) has been used in herbal form for thousands of years to cure cardiovascular diseases [62]. Allitridi is an active constituent that is extracted from* Allium sativum* L. Allicin has antimicrobial and anticancer effects [63], and it lowers blood pressure [64], provides cardiac protection against ischemia/reperfusion insult [65], and also has antiarrhythmic effects. A recent report showed that allitridi can block hKv4.3 channels, and it could also inhibit hKv1.5 channels, hERG channels, and the hKCNQ1/hKCNE1 channels expressed in HEK 293 cells [66]. Other reports show that allitridin impairs the trafficking of hERG channels to reduce the IKr current [67]. Allicin was also proposed to have a role in cardiac conduction that is similar to that of amiodarone, such as calcium channel blockers and IKr and IKs channel blockers [68, 69].

## 8. Compounds

### 8.1. Wenxin-Keli

Wenxin-Keli (WXKL) is a Chinese herbal compound extract developed by Guang'anmen Hospital at the Chinese Academy of Chinese Medical Sciences. WXKL could reportedly increase coronary blood flow, reduce myocardial oxygen consumption, enhance myocardial compliance, improve myocardial hypoxia tolerance, relieve anterior and posterior cardiac loading, and reduce the occurrence of arrhythmia [70]. WXKL includes the following five primary components:* Codonopsis*,* Polygonatum*,* Panax*, nard, and amber. In fact, signal transduction pathways have been a mainstay for cardiovascular therapies for the past 60 years. A meta-analysis showed that, in the aspect of safety, the rate of gastric adverse reactions caused by WXKL is lower than that caused by amiodarone. The calcium/calmodulin-dependent protein kinase II (CaMKII) is a multifunctional serine/threonine kinase and plays a central role in the regulation of intracellular calcium. Dysfunction in CaMKII has been associated with a number of cardiovascular phenotypes, including heart failure and arrhythmia. Studies have reported that WXKL may inhibit the cardiac arrhythmias by regulating the CaMKII signal transduction [71]. A large number of clinical trials have confirmed that WXKL can prolong the AP and block the ICa-L [72]. In present study, we demonstrated that WXKL can inhibit ICa, L, and Ito in a concentration-dependent manner, and it may attenuate ischemia-induced ventricular arrhythmias in rats [73]. Research from Antzelevitch's laboratory demonstrated that WXKL inhibited the fast sodium current (INa) in canine-isolated coronary-perfused preparations through a unique mechanism, and as a result, it may suppress atrial fibrillation (AF) [74].

### 8.2. Shensongyangxin

Shensongyangxin (SSYX) consists of* Panax ginseng*,* Ophiopogon japonicus*, Fructus Corni,* Salvia miltiorrhiza* Radix et Rhizoma, parched semen of Ziziphi Spinosa, Herba Taxilli Chinensis, and so on. The SSYX capsule was approved by the State Food and Drug Administration (SFDA) of China and has been widely used in the treatment of ventricular premature complexes (VPCs) and atrial premature complexes (APCs) in China [75]. Clinical studies have shown that SSYX capsules can effectively treat premature ventricular contraction and alleviate premature ventricular contraction-related symptoms when compared with mexiletine [76]. Basic research shows that SSYX blocks both I(Na) and I(Ca, L), which may contribute to some of its antiarrhythmic effect [77, 78].

## 9. Discussion 

With the development of biological technology and the advent of patch clamp technique, understanding of arrhythmia mechanisms was deeply known. Since the CAST trial in 1989, more and more people realized that single ion channels cannot be effective in the treatment of arrhythmia, the proteins, the genes, and the ion channels in combination would be a new direction for the treatment of cardiac arrhythmias, and more and more genes and proteins related antiarrhythmias were found, including M3 muscarinic acetylcholine receptor (M3-mAChR), the connexin 43 (Cx43), miR-1, miR-133, miR-590, and the calcium/calmodulin-dependent protein kinase II (CaMKII). It was found that many natural drugs through regulation of cell signal transduction pathway can effectively treat the arrhythmias, for example, WXKL and DSS.

With regard to the cardiovascular system, we know that the incidence and the mechanisms of cardiac arrhythmia are a complex process. Heart diseases are always regulated by autonomic nervous systems through their transmitters and modulators, binding to cell surface receptors. Between them, M3-mAChR has been reported for many years. Cholinergic receptors are always divided into muscarinic and nicotinic subtypes. The muscarinic acetylcholine receptor (mAChR) represents a subfamily of G-protein-coupled receptors (GPCRs), which includes M1–M5 subtypes [79]. Many studies confirmed M3-mAChR in the working heart. M3-mAChR can activate a delayed rectifying K+ current IKM3, and it also can participate in cardiac repolarization.

Among other influential factors, there is the Cx43, which is composed of two connexins from the neighboring cardiomyocytes' membrane. It plays a significant role in the cell-to-cell communication between cardiac cells. In ventricle, the major junction protein is Cx43, and it can ensure cardiac electric conduction and electric synchronicity. And the study has demonstrated that Cx43 remodeling may account for intercellular calcium overload, and it is also associated with induction. In fact, intracellular Ca2+ ([Ca2+]*i*), which is known to be related to the early and delayed afterdepolarizations, is directly to be proved that it is associated with the occurrence of arrhythmias of ischemic arrhythmia [80].

Calcium leak, gap junction protein, and autoantibody against ICaL channel were involved in arrhythmogenesis. They provided a theoretical basis for the development of effective antiarrhythmic drugs. Remarkably, as a kind of important RNA regulating gene expression, microRNA (miRNA) was shown to possess antiarrhythmic activities which may prevent cardiac sudden death. Since the discovery of microRNA (miRNA), it is a type of single stranded and noncoding RNAs. It have been identified in humans for 700 miRNAs. Indeed, reportedly, miRNAs can regulate many complex processes in the body, and aberrant expression of various miRNA species has been implicated in many disease states [81]. miR-1, miR-133, and miR-590 regulated the arrhythmia in various types of animal models. On the account of the multiple-gene regulation actions of miRNA, it has the potential to be developed as novel antiarrhythmic target.

The relationship between antioxidant stress and antiarrhythmic effect is considered to play an important role in antiarrhythmic action for some natural compounds [82]. Moreover, the cardiac fibrosis and inflammation also affect cardiac electrophysiological characteristics and arrhythmia [83, 84], but we do not find a natural drug which can via oxidative stress mechanism regulate arrhythmia so far.

Natural drugs have been globally recognized for having broad clinical prospects because of their advantages with respect to multiple targets, significant efficacy, and safety. With the development of modern technology, such as the patch clamp and confocal laser, we have assessed a variety of antiarrhythmic Chinese medicines or their effective ingredients. The chemical structures of these effective ingredients are complex, and they include alkaloids, cardiac glycosides, flavonoids, saponins, coumarins, and naphtha, but mostly alkaloids (Table 1). Great progress has been made on the investigation of traditional Chinese medicine compounds. For example, WXKL has been tested effectively for multiple ion channels by patch clamp technique, especially by Professor Alexander Burashnikov's team in the United States at the Utica (New York) Masonic Medical Laboratory, which confirmed that WXKL can selectively inhibit the atrial sodium channel current, and therefore it can effectively inhibit atrial fibrillation. This compound caused extensive concern in Western medicine, yielding eleven published papers. This compound is one of the clearest representatives of progress in studying the pharmacological mechanism of arrhythmia treatments.

However, treating arrhythmias with natural drugs has the following limitations. First, the antiarrhythmia mechanism of natural drugs is not clear, and the theoretical conclusions are always deduced. Second, clinical and experimental studies have insufficient samples, there are no clear inclusion or exclusion criteria, and the evaluation is always based on electrocardiography; thus, the results are lacking in corresponding conclusiveness. Third, in studying the antiarrhythmic effects and mechanisms of natural drugs, arrhythmias in animal models are mostly drug-induced arrhythmias, which involve a different approach than using the theoretical perspective of traditional Chinese medicine, which would employ the symptoms of arrhythmia, the mechanisms and study of the disease etiology, and arrhythmia pathogenesis. Therefore, the antiarrhythmic effects of traditional Chinese medicine may be defective.

In summary, we should formulate unified perfect research methods and standards, improve the technology, and clarify the role of each component. Then, we will be able to draw a definitive conclusion about the efficacy and safety of natural drugs on arrhythmia.

## Figures and Tables

**Table 1:** Natural drug therapies with antiarrhythmic properties.

Active ingredients	Chemical structure	Natural drug	Molecular formula	Mechanism of action					State of evidence
				I_K_	I_Ca_	I_Na_	APD	Others	
Alkaloid	Diterpenoid alkaloids	Aconitine	C_34_H_47_NO_11_	Reduction in I_Ks_	+	+	Prolong	—	In vitro/animal models
	Isoquinoline	Berberine	C_20_H_18_NO_4_	Reduction in I_to_	−	−	—	—	In vitro/animal models
		Liensinine	C_37_H_42_N_2_O_6_	Reduction in I_Kr_	+	−	—	—	In vitro/animal models
	Indole alkaloids	Rhynchophylline	C_22_H_28_N_2_O_4_	—	+	−	—	—	In vitro/animal models
	Quinolizidine	Matrine	C_15_H_24_N_2_O_2_·H_2_O	Reduction in I_KM3_	+	−	Prolong	—	Humans-RCT
	Alkaloids	Oxymatrine	C_15_H_24_N_2_O_2_		+	+	—	—	In vitro/animal models
		Sophocarpine	C_2_H_22_N_2_O	Reduction in I_Kr_	+	+	Prolong	—	In vitro/animal models
Glycoside		Ginsenoside Rg1	C_42_H_72_O_14_	Reduction in I_Kr_	−	−	—	—	In vitro/animal models
		Phenylpropanoid glycosides	C_29_H_36_O_15_	Reduction in I_Kr_	+	−	Prolong	—	In vitro/animal models
Flavonoid		Baicalein	C_15_H_10_O_5_	—	−	−	—	Pal-LPC	In vitro/animal models
		Resveratrol	C_14_H_12_O_3_	Reduction in I_Ks_	+	+	Short	—	In vitro/animal models

Terpene		Ginkgolide	C_19_H_21_NO_4_	—	+	−	Short	—	In vitro/animal models
Quinones		Tanshinone* II*A	C_19_H_18_O_3_	Reduction in I_K1_	−	−	—	—	In vitro/animal models
Others		Crataegus	C_20_H_27_NO_11_	Reduction in I_Ks_ and I_Kr_	−	−	Prolong	—	In vitro/animal models
		Danshensu	C_9_H_10_O_5_		−	−	—	Cx43	In vitro/animal models
		Omega-3 fatty acids	—	—	+	+	—	—	Humans-RCT
		Allitridi	C_6_H_10_S_2_O	Reduction in I_Kr_, I_ks_, hKv4.3, hKv1.5, and CNQ1/hKCNE1	−	−	—	—	In vitro/animal models

WXKL				Reduction in I_to_	+	+	Prolong	—	Humans-RCT
SSYX					−	−			In vitro/animal models

Pal-LPC = lysophosphatidylcholine; RCT = randomized controlled trial; Cx43 = gap junctional connexin 43; APD = action potential duration; WXKL = Wenxin-Keli; SSYX = Shensongyangxin.
